# A Novel Steroidogenic Acute Regulatory Protein (StAR) Mutation Causing Adrenal Insufficiency in a Neonate: A Case Report of a Rare Medical Condition

**DOI:** 10.7759/cureus.66080

**Published:** 2024-08-03

**Authors:** Aditi Rawat, Sagar Karotkar, Mahaveer Lakra, Ravi Reddy, Revatdhamma Meshram, Amar Taksande

**Affiliations:** 1 Department of Neonatology, Datta Meghe Institute of Higher Education and Research, Wardha, IND; 2 Department of Pediatrics, Datta Meghe Institute of Higher Education and Research, Wardha, IND

**Keywords:** hyperpigmentation, hyperkalemia, hyponatremia, neonate, star mutation, lipoid congenital adrenal hyperplasia

## Abstract

Congenital lipoid adrenal hyperplasia is a very rare and severe cause of adrenal insufficiency. It occurs due to a mutation of the steroidogenic acute regulatory protein (StAR), disrupting adrenal steroid biosynthesis. Here, we report a case of a three-week-old female infant with vomiting, failure to thrive, electrolyte imbalance, and generalized hyperpigmentation. The hormonal assay and genetic diagnosis confirmed a mutation in the StAR protein, leading to adrenal insufficiency. Appropriate replacement therapy resulted in the resolution of clinical and biochemical abnormalities. This case is being reported for its rare etiology and diagnostic clues. It can guide clinicians to keep adrenal insufficiency as a differential diagnosis in a neonate presenting with hyperpigmentation and electrolyte disturbance to save lives.

## Introduction

Congenital lipoid adrenal hyperplasia (CLAH) is an extremely rare subtype of congenital adrenal hyperplasia (CAH). The first step in the adrenal and gonadal steroid synthetic pathway is blocked, leading to the nonconversion of cholesterol to pregnenolone [1]. The incidence of this genetic disorder is 1 in 250,000 to 1 in 300,000 newborns, and it is more frequent in the Japanese and Korean populations [2]. There is a progressive accumulation of cholesterol in the adrenal gland and the deficient synthesis of glucocorticoids, mineralocorticoids, and androgens culminating in hypoglycemia, electrolyte imbalance, abnormal male external genitalia, and adrenal hypertrophy in the neonate [3]. Here, we present a case of a three-week-old neonate who presented with symptoms of vomiting, hyponatremia, and hyperkalemia due to this rare genetic disorder.

## Case presentation

**Figure 1:**
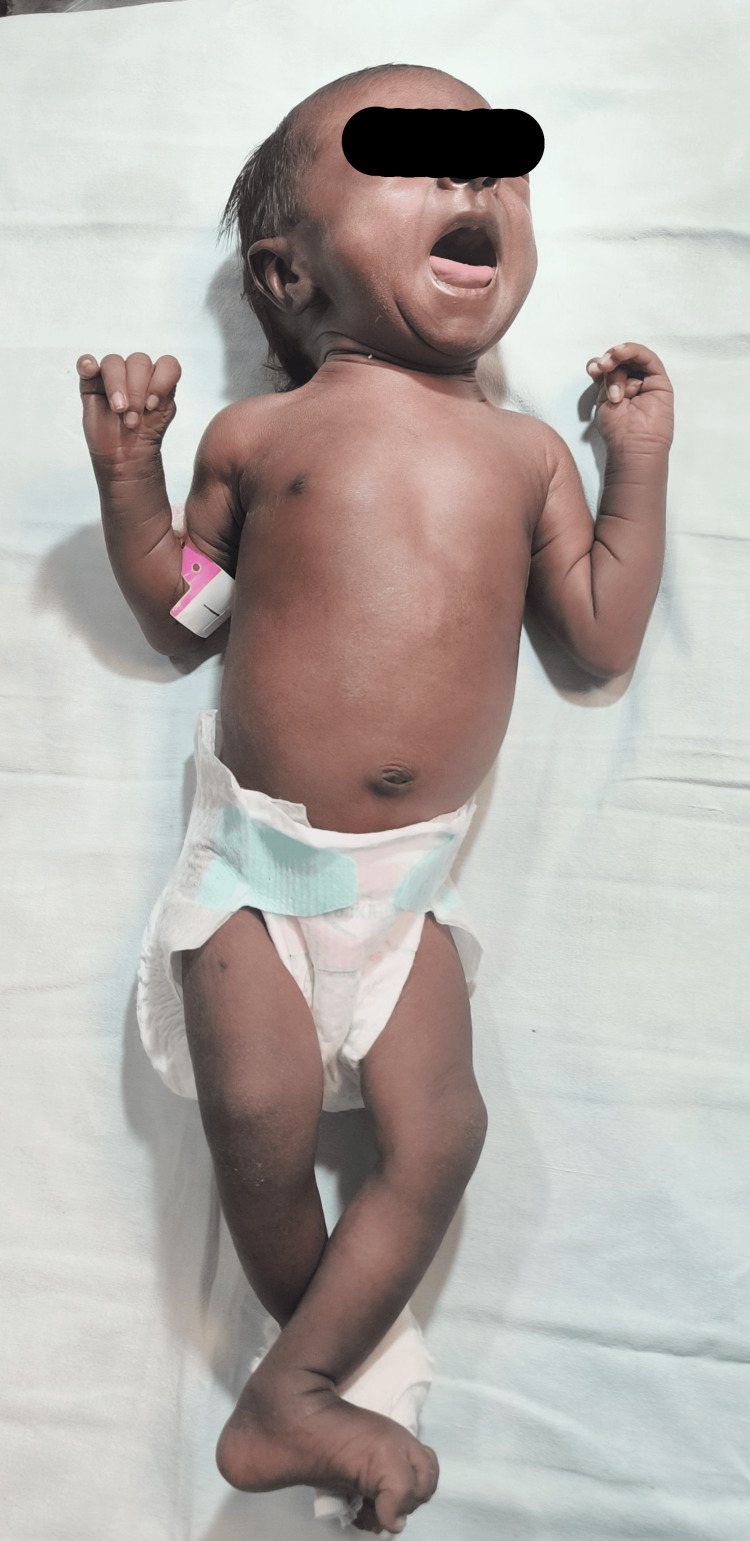
Generalized hyperpigmentation of skin

A female infant with a birth weight of 3 kg was born to a primigravida mother at 37 weeks of gestational age, with no antenatal or postnatal complications. On day 22, the baby was admitted to our hospital with lethargy and vomiting. On examination, the baby was hyporesponsive with a heart rate of 145/minute, respiratory rate of 55/minute, and well palpable pulses. Random blood sugar was 28 mg/dL, for which a bolus of 10% dextrose at 2 mL/kg was given. IV fluids with a glucose infusion rate (GIR) of 6 mg/kg/minute were started. The skin had generalized dark pigmentation, which parents complained was progressively increasing, as skin color was fair at birth, as shown in Figure 1.

**Figure 2:**
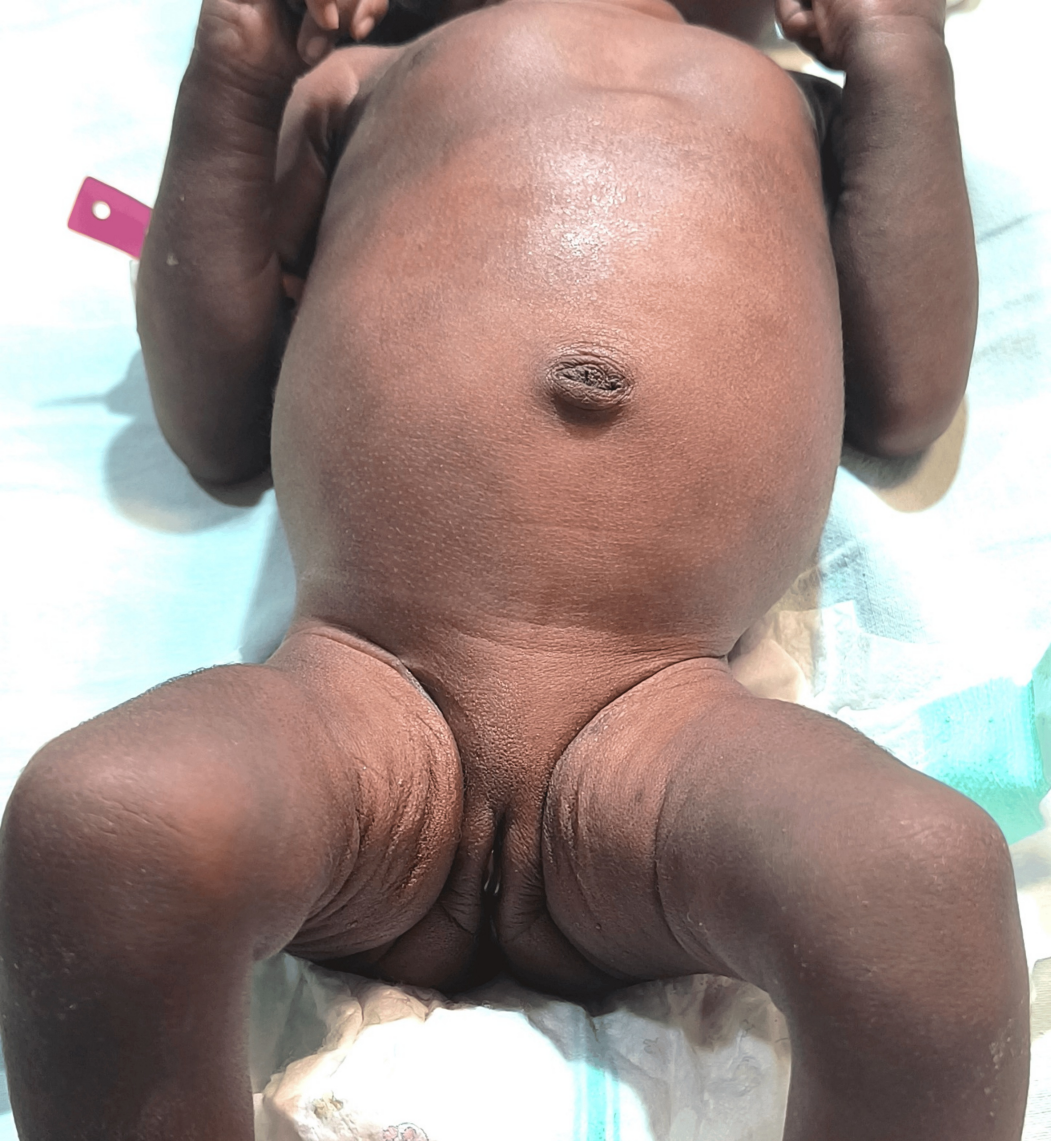
Normal female external genitalia

Normal external female genitalia was present with no palpable abdominal or inguinal mass, as shown in Figure 2.

There was no family history of similar conditions, any endocrine diseases, or neonatal deaths. The infant had failed to thrive as well, with 23% weight loss since birth. Initial investigation ruled out sepsis and meningitis, but hyponatremia and hyperkalemia with acute kidney injury were noted, as shown in Table 1.

**Table 1:** Electrolytes and renal function test reports Normal reference range in neonates: serum sodium: 135-145 mEq/L; serum potassium: 3.5-5.2 mEq/L; serum urea: 5-15 mg/dL; serum creatinine: 0.54-1 mg/dL

Day of life		Serum sodium (mEq/L)	Serum potassium (mEq/L)	Serum urea (mg/dL)	Serum creatinine (mg/dL)
Day 22	Morning	117	6.9	98	1.7
	Evening	124	6.5	81	1.4
Day 23	Morning	120	6.5	65	1.1
	Evening	122	6	42	1
Day 24	Morning	121	5.8	26	0.7
	Evening	122	5.5	-	-
Day 25		121	5.6	7	0.3
Day 27		138	4.6	-	-
Day 28		140	4.2	-	-
Day 32 (on discharge)		142	4.2	14	0.5
On follow-up		139	4	10	0.3

Slow dehydration correction was administered over 72 hours, and electrolytes were monitored 12 hours a day. Renal functions showed gradual improvement, but hyponatremia and hyperkalemia persisted even after sodium supplementation and administration of potassium-free fluids. There were episodes of intermittent asymptomatic hypoglycemia, mandating an increase of GIR up to 10 mg/kg/minute.

Considering the constellation of features like dark pigmentation of the skin, hyponatremia, hyperkalemia, hypoglycemia, and failure to thrive, a diagnosis of adrenal insufficiency was made. Additional hormonal levels are summarized in Table 2.

**Table 2:** Hormone assay

Investigation	Patient’s level	Normal range	Interpretation
Adrenocorticotropic hormone	1,045 pg/mL	<60 pg/mL	High
Aldosterone	4.47 ng/dL	20-790 ng/dL	Low
17-Hydroxyprogesterone	1.19 ng/dL	<250 ng/dL	Low
Cortisol	0.72 mcg/dL	2.8-23 mcg/dL	Low

Ultrasound of the abdomen showed bilaterally enlarged adrenal glands and the presence of Müllerian structures. On day 25, the infant was started on a standard regimen of hydrocortisone at 20 mg/m^2^/day, fludrocortisone at 0.1 mg/day, and sodium supplementation at 3 mEq/kg/day. There was a gradual improvement toward normalization of sodium and potassium, as shown in Table 1. The baby became euglycemic and was able to consume full feeds.

Given multiple episodes of hypoglycemia, an MRI of the brain was done, which showed T2 fluid-attenuated inversion recovery hyperintensities in the occipital region with diffusion restriction, mostly due to hypoglycemic insult to the brain. A genetic study with targeted gene sequencing was done, which gave a final diagnosis of lipoid CAH (LCAH) due to a homozygous mutation of the steroidogenic acute regulatory protein (StAR) gene with autosomal recessive inheritance.

The baby was discharged by day 10 on continued oral hydrocortisone, fludrocortisone, and sodium supplements. The parents were explained genetic counseling before the next pregnancy. On follow-up, after two weeks of discharge, the baby was euglycemic with an adequate weight gain of 20 mg/kg/day and normal electrolytes, as depicted in Table 1.

## Discussion

CLAH is an autosomal recessive disorder caused by a mutation in the StAR gene. The function of StAR is the transport of cholesterol from the outer membrane to the inner membrane of mitochondria so that it can be cleaved into pregnenolone [4]. As a result, further hormone biosynthesis does not occur in the adrenals, leading to a myriad of clinical manifestations. The symptomatology, age of onset, and severity of LCAH varied significantly in cases reported in the literature except for genital phenotype, which is similar in both sexes [5].

The deficiency of glucocorticoids manifests as hypoglycemia with low cortisol levels, as was seen in our case. The mineralocorticoid deficiency is depicted by low aldosterone levels with hyponatremia and hyperkalemia, which was refractory to general electrolyte correction measures in our case, like sodium supplementation and potassium-free fluid. Massive adrenal enlargement is classically seen in LCAH, but some cases have also reported normal or small adrenal gland size [6]. The external genital was of a normal female, and the ultrasound confirmed the presence of Müllerian structures, indicating the female gender. Gender assignment is crucial in CLAH, as neonates with XY karyotypes can have female external genitalia due to the absence of testosterone [7].

Generalized hyperpigmentation served as an important clue for diagnosis. Due to the absence of negative feedback by adrenal hormones, adrenocorticotropic hormone (ACTH) secretion remains unchecked from the anterior pituitary, which causes hyperpigmentation by complementing the increased activity of melanocyte-stimulating hormone [8].

In the most common form of CAH, 21-hydroxylase deficiency, 17-hydroxyprogesterone levels are high, but in our case, it was low, which was a differentiating point. Low levels of 17-hydroxyprogesterone are seen in lipoid adrenal hyperplasia, 17,20 lyase deficiency, and 17-alpha-hydroxylase enzyme deficiency [9].

Treatment for LCAH aims to correct the deficiency in cortisol and aldosterone secretion and suppress ACTH overproduction [10]. In our case, the baby responded very well clinically and biochemically to hydrocortisone and fludrocortisone. Lifelong replacement therapy is needed in such cases. LCAH, being an autosomal recessive disorder, has a 25% chance of recurrence; hence, appropriate antenatal counseling should be done in future pregnancies.

## Conclusions

LCAH is an extremely rare form of CAH that can be missed in newborn screening due to low 17-hydroxyprogesterone levels. Generalized hyperpigmentation with hyponatremia and hyperkalemia should alert the clinician to look for adrenal insufficiency rather than providing routine symptomatic therapy. If undiagnosed, this disease if untreated can turn fatal as the child may go into an adrenal crisis. Thus, clinicians must be very vigilant as early diagnosis and hormonal replacement therapy can be lifesaving for such babies.
